# From cervix to multisite: Detection of lower genital tract lesions in a 10-year cross-sectional colposcopy clinic study

**DOI:** 10.1371/journal.pone.0338489

**Published:** 2025-12-18

**Authors:** Zhongyue Yan, Chenyao Guo, Yao Liu, Jing Li, Quanlong Lu, Ruifang Li, Bo Feng, Chen Wang, Chenju Li, Lei Shi, Tian Feng, Zhilian Wang, Ruimei Feng

**Affiliations:** 1 Department of Epidemiology, School of Public Health, Shanxi Medical University, Taiyuan, China; 2 MOE Key Laboratory of Coal Environmental Pathogenicity and Prevention, Shanxi Medical University, Taiyuan, China; 3 Research Center for Environmental Pollution and Major Chronic Diseases Epidemiology, Shanxi Medical University, Taiyuan, China; 4 Department of Obstetrics and Gynecology, the Second Hospital of Shanxi Medical University, Taiyuan, China; 5 Department of Pathology, the Second Hospital of Shanxi Medical University, Taiyuan, China; University of Catania, ITALY

## Abstract

**Objective:**

To evaluate detection rates of multisite lesions (cervical, vaginal, vulvar) among women attending colposcopy clinics.

**Methods:**

Our cross-sectional study included 20,486 patients between 2014 and 2023 in Shanxi China. Detection rates for cervical, vaginal, and vulvar lesions were retrospectively analyzed across strata by HPV status, cytological diagnosis, and clinical manifestations (vaginal bleeding/discharge). Multinomial logistic regression was applied to calculate odds ratios for high-grade lesions and squamous cell carcinoma (SCC).

**Results:**

High-risk HPV (hr-HPV) infection was detected in 16,636 of 20,486 women (81.2%), and 9,137 (44.6%) had ASC-US+ cytology. Following cervical lesion detection on histopathology among hr-HPV-positive women (CIN2/3: 19.9%; SCC: 5.3%; AIS/ADC: 0.4%), additional lesions were identified at other anatomical sites: vaginal lesions (VaIN2/3: 3.6%; SCC: 1.1%; AIS/ADC: 0.04%) and vulvar lesions (VIN2/3: 0.4%; SCC: 0.1%) were further identified. Overall, 21.6% of hr-HPV-positive women exhibited high-grade lesions (CIN/VaIN/VIN2/3), with 5.6% demonstrating multi-focal SCC and 0.4% showing AIS/ADC. Stratified analysis revealed that patients even with negative HPV or cytology result still had relative high detection rate of high-grade lesions. Among these HPV-negative women, those reporting vaginal bleeding/discharge carried an elevated risk, with 3.2% having high-grade lesions and 10.5% having SCC. The integrative examination combining hr-HPV, cytology, and vaginal bleeding/discharge identified 3,753 high-grade lesions and 1,154 cancers.

**Conclusion:**

Integrating the assessment of hr-HPV testing, cytology, and clinical symptoms (e.g., vaginal bleeding or discharge) could help finding more cases of multisite lesions (cervical, vaginal, vulvar). This is especially important for some high-risk women, including those who visit the colposcopy clinics, and more attention should be paid to the multisite examination.

## 1 Introduction

Cervical cancer, a major malignancy of the lower genital tract, ranks as the fourth most frequently diagnosed cancer and the fourth leading cause of cancer death among women globally [1]. However, vaginal and vulvar cancers are relatively rare malignancies of the lower genital tract; the former accounts for 1% to 2% of gynecological malignancies, while the latter accounts for approximately 4% of all genital cancers. These cancers remain under-recognized in both public awareness and scientific research [2,3]. Multiple European studies reported a rising incidence of vulvar cancer in younger women, potentially driven by human papillomavirus (HPV) infection [4]. HPV infection was well-established to be associated with cervical, vaginal, and vulvar cancers [5–7]. Owing to their shared urogenital sinus origin and anatomical continuity, HPV-induced precancerous lesions may occur synchronously across the cervix, vagina, and vulva [8]. Cervical cancer was considered nearly completely preventable through HPV vaccination and effective screening [1]. Although cervical screening focuses on preventing cervical cancer, the examination may also incidentally note visible vaginal or vulvar lesions.

Common cervical cancer screening methods include cytology and high-risk HPV (hr-HPV) testing, which have proven effective in reducing cervical cancer mortality through precancerous lesion detection [9]. China's cervical cancer screening strategy has progressively shifted from cytology-based to HPV-based testing [10]. Previous studies indicated that while cytology-based screening reduces cervical cancer incidence in high-resource settings [11], it identified only 50% to 70% of high-grade cervical intraepithelial neoplasia (CIN2+) [12]. In contrast, HPV testing can identify a greater number of precancerous lesions, yet it also raises concerns about over-referral to colposcopy and psychological distress in HPV-positive women without significant histopathology [12,13]. Studies in Europe and North America have demonstrated that co-testing provided a high degree of reliability in ruling out CIN3+ over 5-year intervals [14], and current guidelines increasingly recommend co-testing (cytology plus HPV testing) [15]. But evidence regarding co-testings applicability in China and other Asian populations remains limited.

While current research focused predominantly on cervical lesions, the incremental yield of concurrent vaginal/vulvar pathology detection – particularly through symptom-directed assessment – remains under-explored. This study aimed to evaluate findings from a comprehensive colposcopy-based assessment, quantifying the added diagnostic value of multisite examination and determining the predictive utility of vaginal bleeding for high-risk lesions beyond cervical lesions.

## 2 Materials and methods

### 2.1 Study design and participants

The cross-sectional study protocol was approved by the Human Research Ethics Committee of the Second Hospital of Shanxi Medical University (2023 YX No. 350) in Taiyuan, China, informed consent was waived by the Ethics Committee. Data on personal identification were anonymized and de-identified before analysis.

We included women attending their first colposcopy visit between 01/01/2014 and 31/12/2023 with at least one of the following indications: [1] Abnormal cervical testing results: a.positive for HPV16/18 or other persistent hr-HPV types; b.abnormal cytology, including atypical squamous cells-cannot exclude HSIL (ASC-H), low-grade squamous intraepithelial lesion (LSIL) or worse, or atypical squamous cells of undetermined significance (ASC-US) with a positive HPV test; [2] Suspicious clinical findings (e.g., visible cervical lesions, unexplained vaginal bleeding/discharge). [3] HPV-related vulvar or vaginal squamous lesions. [4] Post-treatment follow-up for precancerous lesions of the lower reproductive tract. All eligible participants underwent colposcopy examination. Biopsies were taken only from sites with visible abnormalities; if no lesion was present, no random biopsy was performed. In cases of multiple abnormal-appearing areas, a separate biopsy was obtained from each distinct site for further histopathological confirmation. Data access for research purposes and subsequent analysis commenced in February 2024.

Data on demographics, menopausal status, hysterectomy history, lower genital tract manifestations, HPV genotyping, cytology, and histopathology were retrospectively extracted from clinical records. After all data were input using EpiData software, we excluded patients with missing data on colposcopy ID, age, HPV results, or cytology testing result. Furthermore, cases were considered to have incomplete data and were excluded from analysis if they presented with positive colposcopic findings but lacked corresponding pathological results, and met any of the following criteria: [1] Negative cytology or ASC-US with hr-HPV infection; [2] ASC-H, LSIL, or higher-grade cytology; [3] Hysterectomy with cytological abnormalities or hr-HPV positivity. The final analytical sample comprised 20,486 participants, detailed information were displayed in the study flowchart (S1 Fig).

### 2.2 Cytological diagnosis and HPV genotyping testing

Cervical cell sample was collected using the ThinPrep kit, cytological testing was performed according to the manufacturer's protocol (Hologic Medical Technologies Co., Beijing, China; http://www.hologic.com). Cytological diagnosis was performed by certified pathologists using the Bethesda System (2014) when having no the HPV status. Cases with a diagnosis of ASC-US or worse underwent a blinded review by a senior cytopathologist. 10% sampling cases with the negative cytological result were reviewed by another pathologist for quality control.

HPV genotyping testing was conducted using HybriMax HPV Geno-Array kit (Hybribio Biotechnology Limited Corp., Chaozhou, China) with flow-through hybridization and gene-chip methods, according to the manufacturer's instructions. The manufacturer reports that the assay has a sensitivity of 99.5% and a specificity of 97.7% [16]. The HPV Geno-Array could detect 21 HPV types, including 15 hr-HPV types (16,18,31,33,35,39, 45, 51, 52, 53, 56, 58, 59, 66, and 68) and 6 low-risk HPV (lr-HPV) types (6,11,41,42, 44, and 81) [17]. Hr-HPV infection was defined to having one or more hr-HPV genotypes (including cases with coexisting lr-HPV types); lr-HPV infection was defined to only having lr-HPV genotypes, with no hr-HPV types present.

### 2.3 Cervical, vaginal and vulvar histopathological diagnosis

All histopathological diagnoses were performed according to the World Health Organization (WHO) Classification of Female Genital Tumours (5th edition, 2020) [18] and the LAST consensus guidelines (2020 updates) [19]. To ensure diagnostic consistency, all histopathology slides were independently assessed by two specialist gynecological pathologists. Any diagnostic discrepancies were then resolved through a joint review and discussion until a consensus was reached.

Histopathological diagnosis of cervical lesions included benign cervical inflammatory response (cervicitis), cervical intraepithelial neoplasia (CIN) 1 (CIN1), 2 (CIN2) or 3 (CIN3), squamous cell carcinoma (SCC), adenocarcinoma in situ or adenocarcinoma (AIS/ADC). Vaginal lesions included benign vaginal inflammatory responses (vaginitis), low-grade VaIN (VaIN1), high-grade VaIN (VaIN2 and VaIN3), SCC and AIS/ADC of vagina. Vulvar lesions included vulvitis, low-grade VIN (VIN1), high-grade VIN (VIN2 and VIN3), and SCC of vulva.

### 2.4 Statistical analysis

Distribution differences of cervical, vaginal, or vulvar lesions were compared using the Pearson's Chi-square test or Fisher's exact test across the following variables: age groups (15–24, 25–34, 35–44, 45–54, 55–64, or ≥ 65 years), menopausal status (yes or no), history of hysterectomy (yes or no), clinical manifestations (none, itching, depigmentation, masses/vegetations, vaginal bleeding/discharge, abnormal vaginal discharge, others, or unknown), HPV infection status (negative, hr-HPV infection, lr-HPV infection, HPV infection with unsuccessful typing) and cervical cytological diagnosis (Negative for intraepithelial lesion or malignancy, NILM; ASC-US; ASC-H; LSIL; High-grade squamous intraepithelial lesion, HSIL; SCC; Atypical glandular cells/adenocarcinoma in situ/adenocarcinoma, AGC/AIS/ADC).

Univariate multinomial logistic regression was used to calculate the odds ratios (ORs) and corresponding 95% confidence intervals (CIs) for high-grade lesions (CIN2/3, VaIN2/3, or VIN2/3) and SCC, with the pathology-negative or low-grade lesion (CIN1/VaIN1/VIN1) group as the reference. The detection rate was used to compare single versus multiple lesion distributions in the cervix, vagina, and vulva under different HPV infection statuses. The Pearson's Chi-square test or Fisher's exact test were used to analyze the distribution of various lesions according to: [1] HPV infection subtypes, [2] cytological diagnosis, [3] presence/absence of vaginal bleeding/discharge among clinical manifestations.

SAS 9.4 software was used for statistical analysis; Venny 2.1 was used for Venn diagram generation (https://bioinfogp.cnb.csic.es/tools/venny/). A two-tailed *P* < 0.05 was considered statistically significant.

## 3 Results

### 3.1 Distribution of cervical, vaginal or vulvar lesions

Among the 20,486 women, their average age was 46.3 years. 7,663 (37.4%) were diagnosed with cervicitis or vaginitis or vulvitis, 7,846 (38.3%) with CIN1 or VaIN1 or VIN1, 3,789 (18.5%) with CIN2/3 or VaIN2/3 or VIN2/3, 1,087 (5.3%) with SCC at cervix, vagina or vulva, and 101 (0.5%) with AIS/ADC at cervix or vagina.

The distribution of various cervical, vaginal or vulvar lesions across the general characteristics and clinical features was shown (Table 1). The older patients had higher detection rate of cervical/vulvar/vaginal SCC (1.0% at 15–24 years vs. 19.6% at ≥ 65 years) and AIS/ADC (0 vs. 1.4%) than the younger, with a significant increasing trend of prevalence of the higher-grade lesions with age (*P* < 0.001). Postmenopausal women exhibited significantly higher detection rates of both SCC (10.6% vs. 3.1%) and AIS/ADC (0.8% vs. 0.4%) compared to premenopausal women (*P* < 0.001). Women without a history of hysterectomy had a higher prevalence of CIN2 + /VaIN2 + /VIN2+ than those who had undergone the procedure. Women with vaginal bleeding/discharge (the most common symptoms) had the highest detection rate of SCC (23.3%), and women having masses/vegetations also had relatively higher detection of SCC (7.8%) than women without any symptoms or having itching or depigmentation.

**Table 1:** Characteristics of patients across various cervical, vaginal and vulvar lesions.

Characteristics	Cervicitis or vaginitis or vulvitis	CIN1 or VaIN1 or VIN1	CIN2/3 or VaIN2/3 or VIN2/3	SCC at cervix, vagina or vulva	AIS/ADC at cervix or vagina	ORs and 95% CIs for CIN2/3 or VaIN2/3 or VIN2/3	ORs and 95% CIs for SCC
	N = 7663	N = 7846	N = 3789	N = 1087	N = 101		
	N (%)						
**Age, years** (mean±std)*	45.8 ± 11.1	46.0 ± 11.1	45.3 ± 10.9	55.2 ± 11.7	53.3 ± 11.8		
15-24	117 (38.6)	138 (45.5)	45 (14.9)	3 (1.00)	0	Ref	Ref
25-34	1193 (38.9)	1210 (39.5)	608 (19.8)	48 (1.6)	5 (0.2)	1.43 (1.03, 1.99)	1.70 (0.53, 5.49)
35-44	2244 (38.9)	2115 (36.7)	1230 (21.3)	154 (2.7)	20 (0.4)	1.60 (1.16, 2.21)	3.00 (0.95, 9.47)
45-54	2451 (38.1)	2508 (39.0)	1124 (17.5)	312 (4.8)	32 (0.5)	1.28 (0.93, 1.78)	5.35 (1.70, 16.77)
55-64	1279 (34.2)	1500 (40.1)	599 (16.0)	338 (9.0)	27 (0.7)	1.22 (0.88, 1.70)	10.33 (3.29, 32.42)
≥65	379 (32.0)	375 (31.6)	183 (15.4)	232 (19.6)	17 (1.4)	1.38 (0.96, 1.96)	26.14 (8.30, 82.34)
Menopause status*							
No	5530 (38.4)	5549 (38.5)	2848 (19.8)	442 (3.1)	50 (0.4)	Ref	Ref
Yes	2133 (35.2)	2297 (37.9)	941 (15.5)	645 (10.6)	51 (0.8)	0.83 (0.76, 0.90)	3.65 (3.22, 4.14)
History of hysterectomy*							
No	7356 (37.2)	7544 (38.2)	3720 (18.8)	1057 (5.4)	97 (0.5)	Ref	Ref
Yes	307 (43.1)	302 (42.4)	69 (9.7)	30 (4.2)	4 (0.6)	0.45 (0.35, 0.58)	0.69 (0.48, 1.01)
**Clinical manifestation**							
None*	3905 (31.8)	5723 (46.7)	2376 (19.4)	225 (1.8)	34 (0.3)	Ref	Ref
Itching*	215 (63.6)	92 (27.2)	25 (7.4)	6 (1.8)	0	0.34 (0.23, 0.52)	0.74 (0.33, 1.69)
Depigmentation	19 (51.4)	15 (40.5)	3 (8.1)	0	0	0.40 (0.12, 1.31)	–
Masses/vegetations*	217 (58.5)	90 (24.3)	34 (9.2)	29 (7.8)	1 (0.3)	0.45 (0.32, 0.65)	3.96 (2.65, 5.92)
Vaginal bleeding/discharge*	1147 (38.2)	598 (19.9)	505 (16.8)	700 (23.3)	52 (1.7)	1.18 (1.06, 1.32)	16.78 (14.37, 19.59)
Abnormal vaginal discharge*	128 (72.3)	29 (16.4)	18 (10.2)	2 (1.1)	0	0.50 (0.31, 0.82)	0.25 (0.06, 1.02)
Others*	711 (55.8)	325 (25.5)	165 (13.0)	63 (5.0)	10 (0.8)	0.65 (0.55, 0.77)	2.24 (1.68, 2.98)
Unknown*	1369 (44.0)	994 (32.0)	674 (21.7)	68 (2.2)	5 (0.2)	1.16 (1.05, 1.28)	1.20 (0.92, 1.58)

Abbreviation: CIN, cervical intraepithelial neoplasia; VaIN, vaginal intraepithelial neoplasia; VIN, vulvar intraepithelial neoplasia; SCC, squamous cell carcinoma. AIS/ADC, adenocarcinoma in situ or adenocarcinoma; hr-HPV, high risk HPV; lr-HPV, low risk HPV; ORs, Odds ratios; CIs, Confidence intervals.

*An asterisk denotes a statistically significant between-group difference at *P* < 0.05. Significance was assessed by Pearson's chi-square or Fisher's exact test for pathologic characteristics. For non-exclusive clinical manifestations, *P*-values were obtained from tests for individual categories.

Univariate multinomial logistic regression identified several factors significantly were associated with high-grade lesions (CIN2/3, VaIN2/3, or VIN2/3) and SCC. For high-grade lesions, risk was significantly higher in women aged 25–34 years (OR 1.43, 95% CI 1.03–1.99) and 35–44 years (OR 1.60, 95% CI 1.16–2.21) compared with the reference group (15–24 years). Additionally, the presence of vaginal bleeding/discharge was associated with higher risk of high-grade lesions (OR 1.18, 95% CI 1.06–1.32). For SCC, risk increased substantially with age: compared with the 15–24 years reference group, the ORs were 5.35 (95% CI 1.70–16.77) for 45–54 years, 10.33 (95% CI 3.29–32.42) for 55–64 years, and 26.14 (95% CI 8.30–82.34) for ≥ 65 years. In summary, the risk of SCC escalated dramatically with advancing age, particularly beyond 55 years. Furthermore, menopause (OR 3.65, 95% CI 3.22–4.14), presence of masses/vegetations (OR 3.96, 95% CI 2.65–5.92), and vaginal bleeding/discharge (OR 16.78, 95% CI 14.37–19.59) were also strongly associated with increased odds of SCC.

Among the hr-HPV infected women, CIN2/3 or VaIN2/3 or VIN2/3 (21.6%) and SCC (5.6%) predominated, whereas AIS/ADC demonstrated a higher detection rate among the HPV-negative women (1.2% vs.0.4%). Women with higher-grade cytological diagnosis revealed high-grade cervical or vaginal or vulvar lesions, e.g., women with ASC-H had 47.5% CIN2/3 or VaIN2/3 or VIN2/3 and 22.3% SCC; women with HSIL had 56.5% CIN2/3 or VaIN2/3 or VIN2/3 and 28.5% SCC (S1 Table).

### 3.2 Detection of cervical, vaginal and vulvar lesions stratified by HPV infection status

The detection rates of cervical, vaginal and vulvar lesions in patients with different HPV infection status were shown (Table 2). Among hr-HPV positive women, detection rates for high-grade lesions and SCC were highest in the cervix compared to vaginal and vulvar (CIN2/3: 19.9% vs. VaIN2/3: 3.6% vs. VIN2/3: 0.4%; SCC: 5.3% vs. VaSCC: 1.1% vs. VSCC: 0.1%). Notably, detection rates of high-grade lesions and SCC increased progressively from HPV-negative to lr-HPV to hr-HPV for cervical and vaginal lesions, but vulvar lesions were virtually undetected in lr-HPV positive women. Women without HPV infection had 60.6% detection of benign findings and 1.2% AIS/ADC; lr-HPV infected women had 50.7% low-grade lesions (CIN1 or VaIN1 or VIN1); hr-HPV positive women had a higher prevalence of high-grade concurrent lesions (21.6% CIN2/3 or VaIN2/3 or VIN2/3, 5.6% SCC).

**Table 2:** The detection rates of cervical, vaginal and vulvar lesions among women with different HPV infection status.

Pathological results	HPV negative		lr-HPV positive		hr-HPV positive	
	N = 3387		N = 377		N = 16628	
	N	Detection rate (%)	N	Detection rate (%)	N	Detection rate (%)
**Isolated lesions at cervix, vagina and vulva**						
**Cervix**						
Cervicitis/Negative	2216	65.4	183	48.5	6359	38.2
CIN1	891	26.3	166	44.0	6014	36.2
CIN2/3	143	4.2	22	5.8	3307	19.9
SCC	104	3.1	6	1.6	886	5.3
AIS/ADC	33	1.0	0	0	62	0.4
**Vagina**						
Vaginitis/Negative	3026	89.3	301	79.84	13475	81.0
VaIN1	290	8.6	66	17.51	2362	14.2
VaIN2/3	29	0.9	6	1.59	596	3.6
VaSCC	33	1.0	4	1.06	189	1.1
VaADC	9	0.3	0	0	6	0.04
**Vulva**						
Vulvitis/Negative	3258	96.2	357	94.7	16063	96.6
VIN1	105	3.1	20	5.3	490	3.0
VIN2/3	3	0.1	0	0	59	0.4
VSCC	21	0.6	0	0	16	0.1
**Multiple lesions**						
Cervicitis or/and vaginitis or/and vulvitis	2053	60.6	154	40.9	5429	32.7
CIN1 or/and VaIN1 or/and VIN1	1005	29.7	191	50.7	6612	39.8
CIN2/3 or/and VaIN2/3 or/and VIN2/3	151	4.5	25	6.6	3588	21.6
SCC at cervix or/and vagina or/and vulva	139	4.1	7	1.9	937	5.6
AIS/ADC at cervix or/and vagina	39	1.2	0	0	62	0.4

Abbreviation: CIN, cervical intraepithelial neoplasia; VaIN, vaginal intraepithelial neoplasia; VIN, vulvar intraepithelial neoplasia;SCC, squamous cell carcinoma; AIS/ADC, adenocarcinoma in situ or adenocarcinoma; lr-HPV, low risk HPV; hr-HPV, high risk HPV.

**Fig 1:**
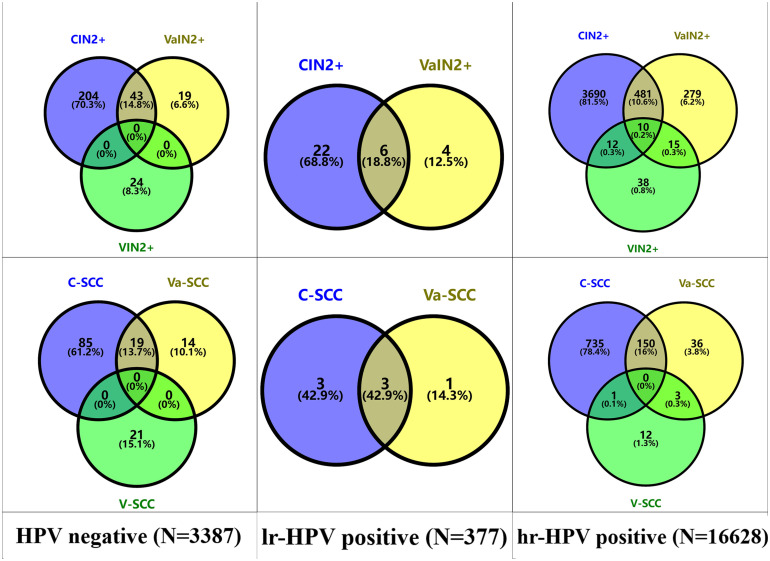
The detection distribution of cervical, vaginal, or vulvar lesions in different HPV infection states. **Abbreviation:** CIN2 + , cervical intraepithelial neoplasia 2 and worse; VaIN2 + , vaginal intraepithelial neoplasia 2 and worse; VIN2 + , vulvar intraepithelial neoplasia 2 and worse; SCC, squamous cell carcinomar; C-SCC, cervical squamous cell carcinoma; Va-SCC, vagina squamous cell carcinoma; V-SCC, squamous cell carcinoma of vulva; lr-HPV, low risk HPV infection; hr-HPV, high risk HPV infection.

The concurrent detection of cervical, vaginal, and vulvar lesions stratified by HPV status was shown in the Venn diagram (Fig 1). Among the HPV-negative women, 43 cases (14.8% of all CIN2 + /VaIN2 + /VIN2 + cases) showed concurrent CIN2+ and VaIN2 + , including 19 SCCs (13.7% of total SCC cases). No VIN2 + was detected among the lr-HPV positive women, 6 cases (18.8% of all CIN2 + /VaIN2 + cases) exhibited co-occurrence of CIN2+ and VaIN2+ with 3 SCCs (42.9% of total SCC cases). Among the hr-HPV positive women, 10 cases (0.2%) had tri-site CIN2 + /VaIN2 + /VIN2+ (no SCC); 481 cases (10.6%) showed CIN2+ and VaIN2 + , including 150 SCCs; 12 cases (0.3%) presented CIN2+ and VIN2+ (1 SCC); 15 cases (0.3%) displayed VaIN2+ and VIN2+ (3 SCCs).

### 3.3 Detection of cervical, vaginal and vulvar lesions stratified by HPV infection status and cytological diagnosis

The detection rates of cervical, vaginal and vulvar lesions stratified by HPV status and cytological diagnosis were shown (Table 3). Among the HPV-negative women, 1,344 (39.7%) had the NILM, demonstrating 2.2% high-grade lesions (30 cases CIN2/3 or VaIN2/3 or VIN2/3), 3.7% SCC (49 cases), and 0.7% AIS/ADC (10 cases). The remaining 2,043 (60.3%) with abnormal cytology result showed high-grade lesion rates of 2.9% for women with ASC-US, 16.1% for ASC-H, 11.6% for LSIL, and 35.6% for HSIL. Rates of histologically diagnosed SCC were 10.7% for ASC-H, 28.9% for HSIL, and 64.3% for SCC cytology.

**Table 3:** The detection rates of cervical, vaginal and vulvar lesions across different cytological diagnosis and HPV infection status.

Cytological diagnosis	Cervicitis or vaginitis or vulvitis	CIN1 or VaIN1 or VIN1	CIN2/3 or VaIN2/3 or VIN2/3	SCC at cervix, vagina or vulva	AIS/ADC at cervix or vagina	Total
	N (%)					
HPV negative*						**3387**
NILM	892 (66.4)	363 (27.0)	30 (2.2)	49 (3.7)	10 (0.7)	1344
ASC-US	1029 (63.8)	481 (29.8)	47 (2.9)	39 (2.4)	16 (1.0)	1612
ASC-H	27 (48.2)	14 (25.0)	9 (16.1)	6 (10.7)	0	56
LSIL	82 (34.0)	125 (51.9)	28 (11.6)	6 (2.5)	0	241
HSIL	14 (13.5)	15 (14.4)	37 (35.6)	30 (28.9)	8 (7.7)	104
SCC	1 (7.1)	1 (7.1)	0	9 (64.3)	3 (21.4)	14
AGC/AIS/ADC	8 (50.0)	6 (37.5)	0	0	2 (12.5)	16
lr-HPV positive*						**377**
NILM	83 (51.9)	73 (45.6)	4 (2.5)	0	0	160
ASC-US	53 (43.4)	60 (49.2)	5 (4.1)	4 (3.3)	0	122
ASC-H	0	2 (50.0)	1 (25.0)	1 (25.0)	0	4
LSIL	17 (22.1)	50 (64.9)	9 (11.7)	1 (1.3)	0	77
HSIL	1 (7.1)	6 (42.9)	6 (42.9)	1 (7.1)	0	14
SCC	0	0	0	0	0	0
AGC/AIS/ADC	0	0	0	0	0	0
hr-HPV positive*						**16628**
NILM	4335 (44.2)	4153 (42.4)	1141 (11.6)	150 (1.5)	25 (0.3)	9804
ASC-US	787 (23.3)	1393 (41.3)	896 (26.6)	281 (8.3)	18 (0.5)	3375
ASC-H	28 (7.5)	58 (15.4)	197 (52.4)	89 (23.7)	4 (1.1)	376
LSIL	249 (12.6)	913 (46.4)	748 (38.0)	57 (2.9)	3 (0.2)	1970
HSIL	25 (2.5)	93 (9.2)	593 (58.8)	292 (28.9)	6 (0.6)	1009
SCC	2 (2.6)	1 (1.3)	7 (9.1)	66 (85.7)	1 (1.3)	77
AGC/AIS/ADC	3 (17.7)	1 (5.9)	6 (35.3)	2 (11.8)	5 (29.4)	17

Abbreviation: CIN, cervical intraepithelial neoplasia; VaIN, vaginal intraepithelial neoplasia; VIN, vulvar intraepithelial neoplasia; SCC, squamous cell carcinoma; AIS/ADC, adenocarcinoma in situ or adenocarcinoma; lr-HPV, low risk HPV; hr-HPV, high risk HPV; NILM, negative for intraepithelial lesion or malignancy; ASC-US, atypical squamous cells of undetermined significance; ASC-H, atypical squamous cells, cannot exclude HSIL; LSIL, Low-grade squamous intraepithelial lesion; HSIL, high-grade squamous intraepithelial lesion; AGC/AIS/ADC, atypical glandular cells/adenocarcinoma in situ/adenocarcinoma.

*An asterisk denotes a statistically significant between-group difference at *P* < 0.05. Significance was assessed by Pearson's chi-square or Fisher's exact test.

Of lr-HPV positive women, 160 (42.4%) with the NILM exhibited 2.5% high-grade lesions (4 cases), with no SCC or AIS/ADC detected. Among 217 abnormal cytology result, 4.1% high-grade lesions were observed for women with the ASC-US (5 cases), 25.0% for the ASC-H (1 cases), 11.7% for the LSIL (9 cases), and 42.9% for the HSIL (6 cases).

Among the hr-HPV positive women, 9,804 (59.0%) with the NILM showed 11.6% high-grade lesions, 1.5% SCC, and 0.3% AIS/ADC. Detection rate of high-grade lesion was 26.6% for women with ASC-US, 52.4% for ASCH, 38.0% for LSIL, and 58.8% for HSIL. Regarding cases with the cytological result of SCC, 85.7% cases were subsequently diagnosed by histopathology.

### 3.4 Detection of cervical, vaginal and vulvar lesions additionally stratified by vaginal bleeding/discharge

Detection rates of cervical, vaginal, and vulvar lesions, stratified by HPV status, cytological diagnosis, as well as vaginal bleeding/discharge, are detailed in Table 4. Regardless of HPV status or cytological diagnosis, there was no significant difference of the detection of CIN2/3 or VaIN2/3 or VIN2/3 between women having and without the vaginal bleeding/discharge symptoms, but the difference of the detection of SCC and AIS/ADC was obvious.

**Table 4:** The distribution of cervical, vaginal and vulvar lesions across the HPV infection status, cytological diagnosis and vaginal bleeding/discharge.

	Cervicitis or vaginitis or vulvitis	CIN1 or VaIN1 or VIN1	CIN2/3 or VaIN2/3 or VIN2/3	SCC at cervix, vagina or vulva	AIS/ADC at cervix or vagina	Total
	N (%)					
**HPV infection status**						
HPV negative*						**3387**
No clinical manifestations	1092 (55.5)	716 (36.4)	114 (5.8)	34 (1.7)	13 (0.7)	1969
Vaginal bleeding/discharge	485 (63.7)	149 (19.6)	24 (3.2)	80 (10.5)	23 (3.0)	761
Others	476 (72.5)	140 (21.3)	13 (2.0)	25 (3.8)	3 (0.5)	657
lr-HPV positive*						**377**
No clinical manifestations	100 (36.6)	149 (54.6)	22 (8.1)	2 (0.7)	0	273
Vaginal bleeding/discharge	24 (54.6)	17 (38.6)	1 (2.3)	2 (4.6)	0	44
Others	30 (50.0)	25 (41.7)	2 (3.3)	3 (5.0)	0	60
hr-HPV positive*						**16628**
No clinical manifestations	4056 (31.1)	5817 (44.6)	2892 (22.2)	255 (2.0)	26 (0.2)	13046
Vaginal bleeding/discharge	637 (29.2)	428 (19.6)	476 (21.8)	615 (28.2)	29 (1.3)	2185
Others	736 (52.7)	367 (26.3)	220 (15.8)	67 (4.8)	7 (0.5)	1397
hr-HPV positive or having vaginal bleeding/discharge*						**20486**
No	1724 (56.7)	1064 (35.0)	172 (5.7)	65 (2.1)	16 (0.5)	3041
Yes	5939 (34.0)	6782 (38.9)	3617 (20.7)	1022 (5.9)	85 (0.5)	17445
**Cytological diagnosis**						
NILM*						**11349**
No clinical manifestations	3642 (42.5)	3905 (45.6)	947 (11.1)	61 (0.7)	10 (0.1)	8565
Vaginal bleeding/discharge	825 (56.0)	368 (25.0)	150 (10.2)	110 (7.5)	20 (1.4)	1473
Others	857 (65.4)	333 (25.4)	87 (6.6)	29 (2.2)	5 (0.4)	1311
ASC-US + *						**9137**
No clinical manifestations	1630 (24.0)	2808 (41.3)	2102 (30.9)	231 (3.4)	29 (0.4)	6800
Vaginal bleeding/discharge	322 (21.1)	230 (15.0)	355 (23.2)	590 (38.6)	32 (2.1)	1529
Others	387 (47.9)	202 (25.0)	148 (18.3)	66 (8.2)	5 (0.6)	808
ASC-US+ or having vaginal bleeding/discharge*						**20486**
No	4499 (45.6)	4238 (42.9)	1034 (10.5)	90 (0.9)	15 (0.2)	9876
Yes	3164 (29.8)	3608 (34.0)	2755 (26.0)	997 (9.4)	86 (0.8)	10610
hr-HPV positive or ASC-US+ or having vaginal bleeding/discharge*						**20486**
No	700 (61.7)	364 (32.1)	36 (3.2)	29 (2.6)	5 (0.4)	1134
Yes	6963 (36.0)	7482 (38.7)	3753 (19.4)	1058 (5.5)	96 (0.5)	19352

Abbreviation: CIN, cervical intraepithelial neoplasia; VaIN, vaginal intraepithelial neoplasia; VIN, vulvar intraepithelial neoplasia; SCC, squamous cell carcinoma; AIS/ADC, adenocarcinoma in situ or adenocarcinoma; lr-HPV, low risk HPV; hr-HPV, high risk HPV; NILM, negative for intraepithelial lesion or malignancy; ASC-US + , atypical squamous cells of undetermined significance and worse.

*An asterisk denotes a statistically significant between-group difference at *P* < 0.05. Significance was assessed by Pearson's chi-square or Fisher's exact test.

Among women with vaginal bleeding/discharge, SCC detection was 10.5% (80/761) for the HPV-negative women and 28.2% (282/2185) for the hr-HPV infection women. Compared with using hr-HPV infection alone, the broader diagnostic criterion of either hr-HPV infection or vaginal bleeding/discharge symptoms identified an additional 29 cases of CIN2/3, VaIN2/3, or VIN2/3; 85 additional SCC cases; and 23 additional AIS/ADC cases.

Among women with vaginal bleeding/discharge, the prevalence of SCC and AIS/ADC varied by cytological diagnosis. Among those with NILM cytology, 7.5% (110) women were diagnosed with SCC and 1.4% [20] with AIS/ADC. In contrast, the rates were significantly higher among women with ASC-US+ cytology, with 38.6% (590) diagnosed with SCC and 2.1% [32] with AIS/ADC. Compared with using ASC-US+ as the sole criterion, incorporating vaginal bleeding/discharge symptoms captured an additional 150 high-grade lesion cases (4.0% of 3,789), 110 SCC cases (10.1% of 1,087), and 20 AIS/ADC cases (19.8% of 101). In addition, taking hr-HPV infection, abnormal cytology result and vaginal bleeding/discharge symptoms together, 3,753 CIN2/3 or VaIN2/3 or VIN2/3 cases, 1,058 SCC cases and 96 AIS/ADC cases were noticed.

## 4 Discussion

Our study was one of the few studies, which described and evaluated the detection of single or concurrent lesions of multiple sites at cervix, vagina and vulva among a large sample of women at the colposcopy outpatient clinic between 2014 and 2023 in Shanxi of China.

### 4.1 Clinical characteristics related to cervical, vaginal or vulvar lesions

In this study, the mean age of women diagnosed with high-grade lesions, SCC, and AIS/ADC was 45.3, 55.3, and 53.3 years, respectively. The detection rates of SCC and AIS/ADC exhibited a pronounced age-associated escalation, particularly in the older age group. Among women aged ≥ 65, SCC and AIS/ADC were 9 times and 4 times higher than in those aged < 45, respectively – a finding corroborated by prior epidemiological studies [20,21]. Both our study and prior research demonstrate that postmenopausal women exhibit significantly elevated risks of SCC compared to premenopausal women [3]. This might be related to the fact that postmenopausal women's aging is accompanied by a decline in immunity. Other studies have shown that the decreased estrogen levels of postmenopausal women may make it easier for HPV to penetrate the thin vaginal epithelium, and menopausal and premenopausal women had a higher risk of prevalent VaIN [22].

In our study, we detected several cases of high-grade lesions, SCC, and AIS/ADC in women with a history of hysterectomy. This clinical finding can be explained by the report from Cao et al. They demonstrated that a history of CIN prior to hysterectomy significantly elevates the risk of high-grade VaIN or vaginal cancer postoperatively [23]. Our study suggested that appearance of vaginal bleeding/discharge and masses/vegetations in the lower genital tract were strongly associated with high-grade lesions, especially with SCC. Clinically, the presence of these symptoms, particularly in postmenopausal women, should raise immediate suspicion for an advanced or invasive lesion, prompting more intensive diagnostic evaluation and expedited management.

Therefore, increased vigilance toward lower genital tract health is warranted for: [1] elderly women, particularly those presenting with postmenopausal vaginal bleeding/discharge; and [2] women who underwent hysterectomy for CIN or cervical cancer.

Our findings highlight that a definitive proportion of lower genital tract cancer cases are HPV-negative, which cautions against sole reliance on hr-HPV testing. Published evidence confirms high-grade cytological abnormalities have determined a greater likelihood of developing into cancer [24]. In this study, we observed the cytological diagnosis showed a high degree of consistency with the pathological diagnosis. Specifically, high-grade cytological abnormalities – particularly ASC-H (70% concordance with neoplasia lesions 2+), HSIL (85% with neoplasia lesions 2+), and SCC cases (90.1% consistency). This high level of agreement reinforces the validity of cytology as a testing method, as it effectively triages patients towards confirmatory diagnosis and timely treatment.

### 4.2 HPV Distribution and Lesion Detection Rates Across Lower Genital Tract Sites

Consistent with other studies, the detection rates of high-grade lesions (cervical, vaginal, and vulvar lesions) and SCC were the highest among hr-HPV positive women, while those diagnosed to be AIS/ADC were HPV negative or hr-HPV positive, This pattern is explained by the fact that HPV-negative cervical cancers are significantly associated with ADC, many of which are distinct sub-types that arise via HPV-independent pathways. This has profound implications for testing method, as a primary HPV testing approach carries a tangible risk of missing these lesions. Consequently, these aggressive tumors may present at a more advanced stage, contributing to their poorer prognosis. This underscores the critical value of cytology as an adjunctive method, which can detect morphological abnormalities even without HPV [25]. Our study supports the view that the hr-HPV infection was the main driving factor for carcinogenesis of lesions at lower genital tract [5–7]. It was worth noting that low-grade lesions (CIN1/VaIN1/VIN1) predominated among the lr-HPV positive population. This finding is consistent with the known biology of low-risk HPV types (such as HPV6/11), which typically cause transient infections and benign proliferations that manifest histologically as condyloma acuminatum [26]. Collectively, preventing HPV-driven lesions necessitates the adoption of genotype-specific prevention approaches.

This study also aligns with existing evidence indicated that CIN frequently coexists with VaIN [27]. In our study, the detection rate of cervical lesions (especially CIN2/3 and SCC) was obviously higher than that of vaginal and vulvar lesions, and the high co-occurrence rate of cervical and vaginal lesions (10.6%) warranted attention, possibly reflecting both the multifocal nature of HPV infection and systemic dysregulation of the loco-regional immune microenvironment [28]. Multiple studies report rising incidence of both HPV-associated and non-HPV-associated vulvar carcinomas [29,30]. Our data align with this trend, demonstrating detectable VIN2/3 and vulvar SCC among HPV-negative and hr-HPV-positive women and noting an apparent increase in these cases in recent years. Given the occurrence of concurrent multifocal involvement and the escalating incidence of vaginal/vulvar pathologies, combined with the anatomic contiguity of the cervix, vagina, and vulva, our findings lend support to the rationale for further investigating the value of integrated management strategies for lower genital tract diseases.

### 4.3 Combined assessment of HPV, cytological examination and specific symptoms

While our data support the use of hr-HPV testing for cervical screening, the observed associations with vaginal and vulvar lesions suggest that its potential utility in a broader lower genital tract screening strategy merits further evaluation. Among women referred to colposcopy clinics, combined hr-HPV testing and cytology demonstrate a high detection rate in identifying potential precancerous or cancerous cases. Notably, among hr-HPV positive patients with HSIL/SCC cytology, the detection rates for high-grade lesions and SCC were considerable indicating a strong association between cytological abnormalities and histologically diagnosed precancerous progression. However, among the hr-HPV positive women with normal cytology, a proportion women were found to have high-grade lesions or SCC, indicating that cytological examination failed to identify all high-risk patients. This discrepancy may stem from sampling issues, the under-detection of specific lesion types, and the biological reality that oncogenic HPV infection can predate observable cytological abnormalities. Furthermore, abnormal cytology identified additional precancer/cancer cases in HPV-negative or lr-HPV-infected women. Crucially, only 39.7% of HPV-negative women in our cohort concurrently showed normal cytology, a finding that may stem either from false-negative HPV results due to low viral load, or, more plausibly, from cytological abnormalities caused by benign inflammatory changes and other non-HPV related pathologies. Thus, relying solely on HPV testing and cytology may be insufficient for comprehensive case detection in colposcopy populations.

Either among the hr-HPV positive or ASC-US+ women, individuals with concurrent vaginal bleeding/discharge exhibited a significantly elevated risk of the high-grade lesions and SCC. However, even among the women with the negative HPV or the NILM, those with vaginal bleeding/discharge still had high detection rate of the SCC cases. This clinical association is biologically plausible, as advanced neoplasia often involves the development of friable, abnormal blood vessels (neoangiogenesis) within the tumor and erosion of the normal epithelial surface, which can lead to contact or spontaneous bleeding. Notably, even among symptomatic HPV-negative patients, 3.3% harbored high-grade lesions and 10.2% had SCC, potentially attributable to HPV testing limitations (e.g., sampling errors or blood-induced dilution) or HPV-independent carcinogenesis [31]; 7.1% of cytology-negative symptomatic patients still had SCC, suggesting cytological sensitivity gaps or occult lesions [32]. This finding demonstrates that assessing vaginal bleeding/discharge symptoms can identify additional precancerous and cancerous lesions, particularly SCC and AIS/ADC.

This aligns with international guidelines prioritizing colposcopy referral for symptomatic patients [33]. Symptoms of vaginal bleeding/discharge should be independently weighted in clinical decision-making, prompting further investigation even with negative cytology.

Our study found that combining HPV testing, cytology and symptom assessment (with at least one positive among the three) could identify more lesion populations compared to a single strategy, including 19.4% of high-grade lesions and 5.5% of SCC.

Despite combining HPV testing, cytology, and symptom assessment, a small proportion of high-grade lesions and cancers remained undetected, highlighting the inherent limitations of current screening strategies. Our findings demonstrate that the current framework can improve the detection of multisite lesions (cervical, vaginal, and vulvar) by integrating primary HPV testing with cytology and systematic symptom assessment. The clinical pathway for the evaluation of lower genital tract lesions was displayed in the study flowchart (S2 Fig).

## 5 Study strengths and limitations

A key strength of our study lies in the simultaneous assessment of cervical, vaginal, and vulvar histopathology in a large sample of women, using consistent diagnostic methods for histology, cytology, and HPV genotyping. All clinical and laboratory procedures were conducted under the hospital's well-documented standard operating protocols. A further strength lies in the stratified evaluation of lesion detection rates (cervical, vaginal, and vulvar) by HPV status and cytology, enabling precise subgroup comparisons.

However, this study also has several limitations. First, the study population was recruited from colposcopy clinics and therefore predominantly comprised patients referred for abnormal results. This inherent selection bias means that the reported lesion detection rates are not representative of the general screening population, or our results indicate the dectection risk of multisite lesions of the high-risk women. Secondly, this study has a retrospective cross-sectional design spanning ten years. While this extended time frame allowed for the observation of long-term trends, no significant changes were found in the age-standardized detection rate of lesions. Additionally, random biopsies were not performed in this study, and therefore, our detection rates are not directly comparable to those derived from biopsy-based screening.

## 6 Conclusion

Integrating the assessment of hr-HPV testing, cytology, and clinical symptoms (e.g., vaginal bleeding or discharge) could help finding more cases of multisite lesions (cervical, vaginal, vulvar). This is especially important for some high-risk women, including those who visit the colposcopy clinics, and more attention should be paid to the multisite examination.

## Supporting information

S1 Fig Flowchart of participant inclusion and exclusion. The flowchart details the stepwise selection process of the 20,486 analyzed participants from the initially enrolled individuals.
(TIF)

S1 Table HPV and cytology findings across various cervical, vaginal, and vulvar lesions. Distribution of HPV infection and cytology results among diagnosed cases of cervical, vaginal, and vulvar lesions.
(DOCX)

S2 Fig Clinical pathway for the evaluation of lower genital tract lesions. The diagram outlines the diagnostic and referral steps following an abnormal finding.
(TIF)

## References

[1] Sung H, Ferlay J, Siegel RL, Laversanne M, Soerjomataram I, Jemal A. Global cancer statistics 2020: GLOBOCAN estimates of incidence and mortality worldwide for 36 cancers in 185 countries. CA: A Cancer J Clin. 2021;71(3):209–49. 10.3322/caac.21660 33538338

[2] Meixner E, Arians N, Bougatf N, Hoeltgen L, König L, Lang K, et al. Vaginal cancer treated with curative radiotherapy with or without concomitant chemotherapy: oncologic outcomes and prognostic factors. Tumori. 2023;109(1):112–20. doi: 10.1177/03008916211056369 34724840 PMC9896533

[3] Dang X, Lu Q, Li J, Li R, Feng B, Wang C, et al. Exploring the potential prompting role of cervical human papilloma virus detection in vulvar lesions: a cross-sectional study in China. Front Oncol. 2024;14. 10.3389/fonc.2024.1353580 PMC10902713 38425337

[4] Faber MT, Sand FL, Albieri V, Norrild B, Kjaer SK, Verdoodt F. Prevalence and type distribution of human papillomavirus in squamous cell carcinoma and intraepithelial neoplasia of the vulva. Inter J Cancer. 2017;141(6):1161–9. 10.1002/ijc.30821 28577297

[5] Galati L, Peronace C, Fiorillo MT, Masciari R, Giraldi C, Nisticò S, et al. Six years genotype distribution of Human Papillomavirus in Calabria region, Southern Italy: a retrospective study. Infect Agent Cancer. 2017;12:43. doi: 10.1186/s13027-017-0154-5 28770002 PMC5531005

[6] Berman TA, Schiller JT. Human papillomavirus in cervical cancer and oropharyngeal cancer: One cause, two diseases. Cancer. 2017;123(12):2219–29. doi: 10.1002/cncr.30588 28346680

[7] Zhai L, Tumban E. Gardasil-9: a global survey of projected efficacy. Antiviral Res. 2016;130:101–9. doi: 10.1016/j.antiviral.2016.03.016 27040313

[8] Robboy SJ, Kurita T, Baskin L, Cunha GR. New insights into human female reproductive tract development. Differentiation. 2017;97:9–22. doi: 10.1016/j.diff.2017.08.002 28918284 PMC5712241

[9] Ronco G, Dillner J, Elfström KM, Tunesi S, Snijders PJF, Arbyn M, et al. Efficacy of HPV-based screening for prevention of invasive cervical cancer: follow-up of four European randomised controlled trials. Lancet. 2014;383(9916):524–32. doi: 10.1016/S0140-6736(13)62218-7 24192252

[10] Rao X, Wang Y-H, Chen R-Z, Wu Q-Q, Zhang X-F, Fu Y-F, et al. Risk-based triage strategy by extended HPV genotyping for women with ASC-US cytology. Ann Med. 2025;57(1):2451183. doi: 10.1080/07853890.2025.2451183 39823191 PMC11749152

[11] Sankaranarayanan R, Thara S, Esmy PO, Basu P. Cervical cancer: screening and therapeutic perspectives. Med Princ Pract. 2008;17(5):351–64. doi: 10.1159/000141498 18685274

[12] Arbyn M, Verdoodt F, Snijders PJF, Verhoef VMJ, Suonio E, Dillner L, et al. Accuracy of human papillomavirus testing on self-collected versus clinician-collected samples: a meta-analysis. Lancet Oncol. 2014;15(2):172–83. doi: 10.1016/S1470-2045(13)70570-9 24433684

[13] McBride E, Tatar O, Rosberger Z, Rockliffe L, Marlow LAV, Moss-Morris R, et al. Emotional response to testing positive for human papillomavirus at cervical cancer screening: a mixed method systematic review with meta-analysis. Health Psychol Rev. 2021;15(3):395–429. doi: 10.1080/17437199.2020.1762106 32449477

[14] Dillner J, Rebolj M, Birembaut P, Petry K-U, Szarewski A, Munk C, et al. Long term predictive values of cytology and human papillomavirus testing in cervical cancer screening: joint European cohort study. BMJ. 2008;337:a1754. doi: 10.1136/bmj.a1754 18852164 PMC2658827

[15] Fontham ETH, Wolf AMD, Church TR, Etzioni R, Flowers CR, Herzig A, et al. Cervical cancer screening for individuals at average risk: 2020 guideline update from the American cancer society. CA Cancer J Clin. 2020;70(5):321–46. doi: 10.3322/caac.21628 32729638

[16] Tao P, Zheng W, Wang Y, Bian M-L. Sensitive HPV genotyping based on the flow-through hybridization and gene chip. J Biomed Biotechnol. 2012;2012:938780. doi: 10.1155/2012/938780 23193367 PMC3495277

[17] International Agency for Research on Cancer. IARC monographs on the evaluation of carcinogenic risks to humans. A review of human carcinogens. Part B: Biological agents. 2012.

[18] WHO Classification of Tumours Editorial Board. Female genital tumours. 5th ed. International Agency for Research on Cancer; 2020.

[19] Darragh TM, Colgan TJ, Cox JT, Heller DS, Henry MR, Luff RD, et al. The lower anogenital squamous terminology standardization project for HPV-associated lesions: background and consensus recommendations from the college of American pathologists and the American society for colposcopy and cervical pathology. J Low Genit Tract Dis. 2012;16(3):205–42. doi: 10.1097/LGT.0b013e31825c31dd 22820980

[20] Arbyn M, Weiderpass E, Bruni L, de Sanjosé S, Saraiya M, Ferlay J, et al. Estimates of incidence and mortality of cervical cancer in 2018: a worldwide analysis. Lancet Glob Health. 2020;8(2):e191–203. doi: 10.1016/S2214-109X(19)30482-6 31812369 PMC7025157

[21] Olsen J, Jørgensen TR, Kofoed K, Larsen HK. Incidence and cost of anal, penile, vaginal and vulvar cancer in Denmark. BMC Public Health. 2012;12(1). 10.1186/1471-2458-12-1082 PMC3546065 23244352

[22] Lu M, Hong X, Liu T, Mai B, Hu G, Sun X. Clinical characteristics and risk factors to high-grade vaginal intraepithelial neoplasia: a single-institution study. BMC Womens Health. 2025;25(1):44. doi: 10.1186/s12905-025-03585-7 39893434 PMC11786430

[23] Cao D, Wu D, Xu Y. Vaginal intraepithelial neoplasia in patients after total hysterectomy. Curr Probl Cancer. 2021;45(3):100687. doi: 10.1016/j.currproblcancer.2020.100687 33309077

[24] McCredie MRE, Sharples KJ, Paul C, Baranyai J, Medley G, Jones RW, et al. Natural history of cervical neoplasia and risk of invasive cancer in women with cervical intraepithelial neoplasia 3: a retrospective cohort study. Lancet Oncol. 2008;9(5):425–34. doi: 10.1016/S1470-2045(08)70103-7 18407790

[25] Lee J-E, Chung Y, Rhee S, Kim T-H. Untold story of human cervical cancers: HPV-negative cervical cancer. BMB Rep. 2022;55(9):429–38. doi: 10.5483/BMBRep.2022.55.9.042 35725012 PMC9537028

[26] Stuqui B, Provazzi PJS, Lima MLD, Cabral ÁS, Leonel ECR, Candido NM, et al. Condyloma acuminata: an evaluation of the immune response at cellular and molecular levels. PLoS One. 2023;18(4):e0284296. doi: 10.1371/journal.pone.0284296 37053156 PMC10101375

[27] Lu M, Luo X, Bu Q, Li P, Luo W, Ji S, et al. Association between low-grade cervical cytology and histological cervical intraepithelial neoplasia concurrent vaginal intraepithelial neoplasia among outpatient colposcopy: a retrospective study. J Obstet Gynaecol Res. 2025;51(4):e16289. doi: 10.1111/jog.16289 40254535

[28] Alizhan D, Ukybassova T, Bapayeva G, Aimagambetova G, Kongrtay K, Kamzayeva N. Cervicovaginal microbiome: physiology, age-related changes, and protective role against human papillomavirus infection. J Clin Med. 2025;14(5). 10.3390/jcm14051521 PMC11900180 40094958

[29] Gebresilasie SF, Asnakew GB, Teklesilasie W. A neglected disease relationship—association of human immunodeficiency viral infection and vulvar cancer: a retrospective, hospital-based, case-control study. Intern J Gynaecol Obstetrics: The Official Organ of the International Federation of Gynaecology and Obstetrics. 2025. 10.1002/ijgo.70289 40488455

[30] Agarossi A, Savasi V, Frangipane C, Parisi F, Agarossi A, Dominoni M, et al. High risk of HPV-related preneoplastic and neoplastic vulvar lesions in women living with HIV. J Low Genit Tract Dis. 2025;29(2):118–22. doi: 10.1097/LGT.0000000000000864 39652424

[31] Höhn AK, Brambs CE, Hiller GGR, May D, Schmoeckel E, Horn L-C. 2020 WHO classification of female genital tumors. Geburtshilfe Frauenheilkd. 2021;81(10):1145–53. doi: 10.1055/a-1545-4279 34629493 PMC8494521

[32] Khunamornpong S, Settakorn J, Sukpan K, Suprasert P, Srisomboon J, Intaraphet S, et al. Genotyping for Human Papillomavirus (HPV) 16/18/52/58 has a higher performance than HPV16/18 genotyping in triaging women with positive high-risk HPV test in Northern Thailand. PLoS One. 2016;11(6):e0158184. doi: 10.1371/journal.pone.0158184 PMC4918932 27336913

[33] Perkins RB, Guido RS, Castle PE, Chelmow D, Einstein MH, Garcia F, et al. 2019 ASCCP risk-based management consensus guidelines for abnormal cervical cancer screening tests and cancer precursors. J Lower Genital Tract Dis. 2020;24(2):102–31. 10.1097/LGT.0000000000000525 PMC7147428 32243307

