# Supplementary figures and images for: From cervix to multisite: Detection of lower genital tract lesions in a 10-year cross-sectional colposcopy clinic study
Source: PLoS One. 2025 Dec 18;20(12):e0338489. doi: 10.1371/journal.pone.0338489 (PMC12714224; doi:10.1371/journal.pone.0338489)

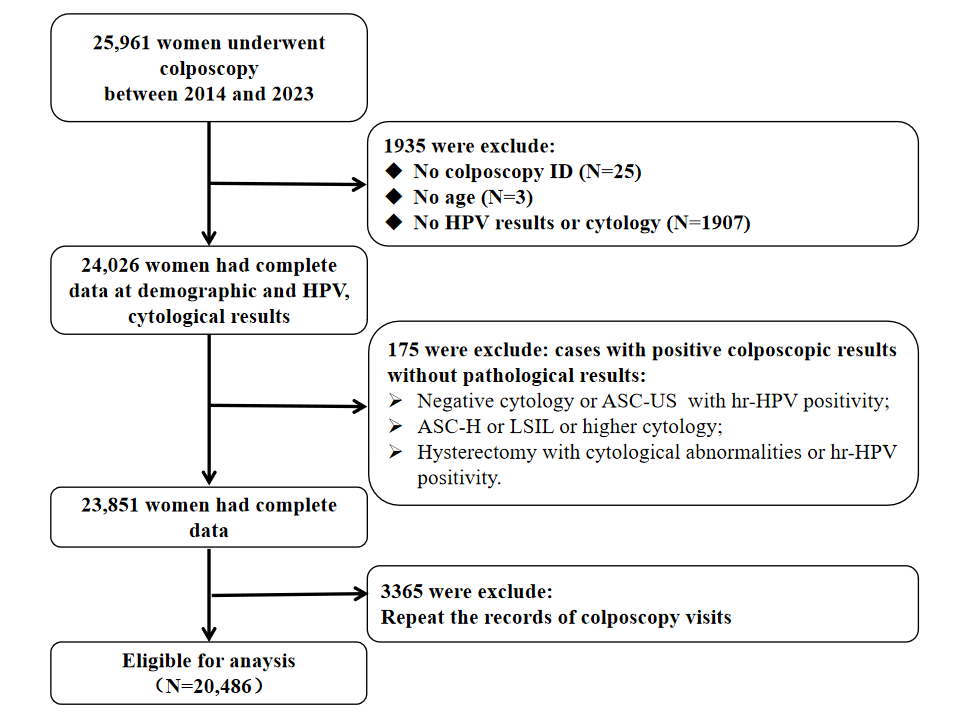

Supplement: S1 Fig — The flowchart details the stepwise selection process of the 20,486 analyzed participants from the initially enrolled individuals. (TIF) [file pone.0338489.s001.tif]

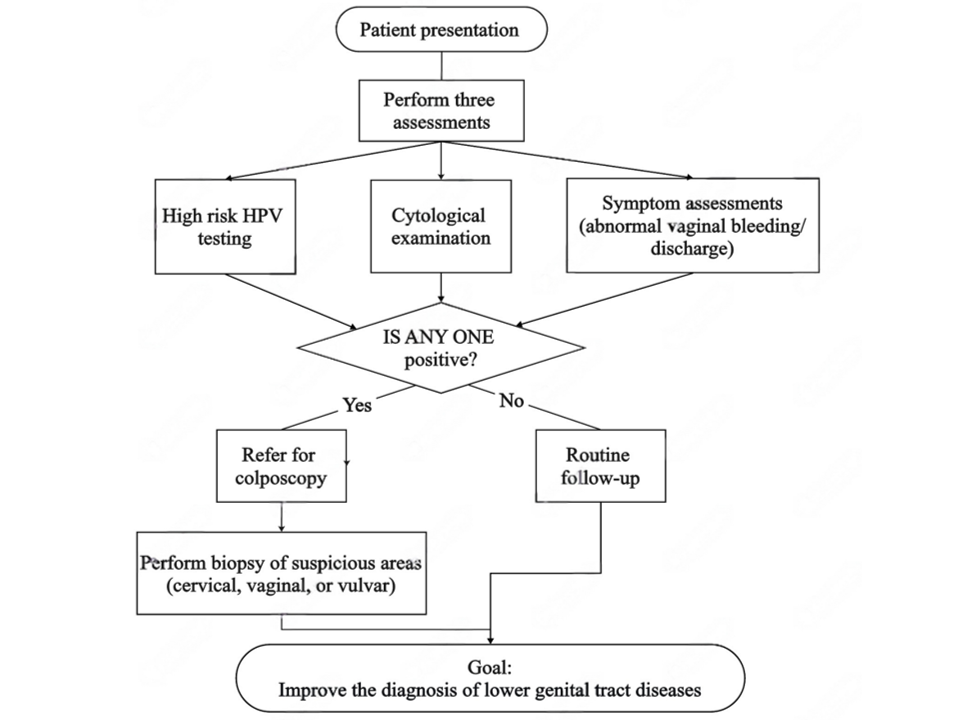

Supplement: S2 Fig — The diagram outlines the diagnostic and referral steps following an abnormal finding. (TIF) [file pone.0338489.s003.tif]
