# Supplementary material for: From cervix to multisite: Detection of lower genital tract lesions in a 10-year cross-sectional colposcopy clinic study
Source: PLoS One. 2025 Dec 18;20(12):e0338489. doi: 10.1371/journal.pone.0338489 (PMC12714224; doi:10.1371/journal.pone.0338489)
Supplement: S1 Table — Distribution of HPV infection and cytology results among diagnosed cases of cervical, vaginal, and vulvar lesions. (DOCX) [file pone.0338489.s002.docx]

**S1 Table. HPV and cytology findings across various cervical, vaginal, and vulvar lesions.**

| **Characteristics** | **Cervicitis or vaginitis or vulvitis** | **CIN1 or VaIN1 or VIN1** | **CIN2/3 or VaIN2/3 or VIN2/3** | **SCC at cervix, vagina or vulva** | **AIS/ADC at cervix or vagina** | **Total** | ***P*** |
| --- | --- | --- | --- | --- | --- | --- | --- |
|  | **N=7663** | **N=7846** | **N=3789** | **N=1087** | **N=101** | **20486** |  |
|  | **N (%)** | | | | |  |  |
| **HPV infection status** | | | | | | | <0.001 |
| Negative | 2053 (60.6) | 1005 (29.7) | 151 (4.5) | 139 (4.1) | 39 (1.2) | 3387 |  |
| hr-HPV Positive | 5429 (32.7) | 6612 (39.8) | 3588 (21.6) | 937 (5.6) | 62 (0.4) | 16628 |  |
| lr-HPV Positive | 154 (40.9) | 191 (50.7) | 25 (6.6) | 7 (1.9) | 0 | 377 |  |
| Unknown HPV genotype | 27 (28.7) | 38 (40.4) | 25 (26.6) | 4 (4.3) | 0 | 94 |  |
| **Cytological diagnosis** | | | | | | | <0.001 |
| NILM | 5324 (46.9) | 4606 (40.6) | 1184 (10.4) | 200 (1.8) | 35 (0.3) | 11349 |  |
| ASC-US | 1878 (36.5) | 1949 (37.9) | 954 (18.6) | 325 (6.3) | 34 (0.7) | 5140 |  |
| ASC-H | 55 (12.5) | 74 (16.8) | 209 (47.5) | 98 (22.3) | 4 (0.9) | 440 |  |
| LSIL | 351 (15.3) | 1092 (47.5) | 788 (34.3) | 64 (2.8) | 3 (0.1) | 2298 |  |
| HSIL | 41 (3.6) | 116 (10.2) | 641 (56.5) | 323 (28.5) | 14 (1.2) | 1135 |  |
| SCC | 3 (3.3) | 2 (2.2) | 7 (7.7) | 75 (82.4) | 4 (4.4) | 91 |  |
| AGC/AIS/ADC | 11 (33.3) | 7 (21.2) | 6 (18.2) | 2 (6.1) | 7 (21.2) | 33 |  |

**Abbreviation:** CIN, cervical intraepithelial neoplasia; VaIN, vaginal intraepithelial neoplasia; VIN, vulvar intraepithelial neoplasia; SCC, squamous cell carcinomar. AIS/ADC, adenocarcinoma in situ or adenocarcinoma; hr-HPV, high risk HPV; lr-HPV, low risk HPV; NILM, negative for intraepithelial lesion or malignancy; ASC-US, atypical squamous cells of undetermined significance; ASC-H, atypical squamous cells, cannot exclude HSIL; LSIL, Low-grade squamous intraepithelial lesion; HSIL, high-grade squamous intraepithelial lesion; AGC/AIS/ADC, atypical glandular cells/adenocarcinoma in situ/adenocarcinoma.
